# Experimental Testing of the Affective Consequences of Nostalgia

**DOI:** 10.11621/pir.2026.0103

**Published:** 2026-03-01

**Authors:** Elina S. Tsigeman, Andrei Y. Sivov, Larisa V. Mararitsa, Maxim V. Likhanov, Ksenia V. Bartseva, Evgenia A. Alenina, Elena L. Soldatova

**Affiliations:** a *Saint Petersburg State University, Russia*; b *HSE University, Saint Petersburg, Russia*; c HSE University, Moscow, Russia

**Keywords:** nostalgia, emotions, positive and negative affect, ambivalent affect, well-being

## Abstract

**Background:**

The inconsistent evidence regarding nostalgia’s affective outcomes suggests that its effects may not be uniform, but contingent on individual characteristics, a possibility that has received limited empirical attention.

**Objective:**

To examine how induced nostalgia influences positive and negative affect, while accounting for age, gender, and overall well-being.

**Design:**

A sample of 122 participants (balanced by gender and across three age groups: 20-35, 36-50, and 51-65 years) completed an Event Reflection Task to elicit either nostalgic memories or recollections of daily life events. Positive and negative affect were measured before and after the nostalgia induction and general well-being was measured after the intervention.

**Results:**

Negative and ambivalent affect decreased after both experimental and control interventions. Gender and age did not moderate the effect of nostalgia induction on affect. Well-being moderated affective outcomes of nostalgia induction, with individuals higher in well-being experiencing greater increases in positive affect and greater decreases in negative and ambivalent affect (co-activation of positive and negative affect) after nostalgia induction.

**Conclusion:**

The emotional impact of nostalgia appears to be independent of age or gender, but affected by individual differences in well-being. These findings help to clarify previous inconsistencies in the literature and suggest that nostalgia may be most emotionally beneficial for those already in a stable emotional state.

## Introduction

Nostalgia is a complex socio-emotional phenomenon. Despite previous negative views on nostalgia as a “form of melancholia” (Peters, 1985), many current studies view nostalgia as “a warm, sentimental longing for the past” (Wildschut et al., 2006). Most of the studies done by the research team of Sedikides, Wildschut, and collaborators demonstrate that nostalgia enhances various aspects of psychological well-being. For example, nostalgia induction (compared to neutral memories) elicited more positive emotions, feelings of social connectedness, and more positive self-esteem (Cox et al., 2015; Reid et al., 2023; Wildschut et al., 2006), and inspired optimism (Cheung et al., 2013). Many authors view nostalgia as a coping mechanism that people can turn to during challenging life events such as migration (Wildschut & Sedikides, 2023), imprisonment (Vartanyan et al., 2024), or the loss of loved ones (Reid et al., 2021). Recently, nostalgia was shown to protect individuals from the negative effect of loneliness during the COVID-19 pandemic lockdown (Zhou et al., 2022), with individuals who engaged in nostalgic experiences exhibiting smaller declines in happiness compared to those who did not. Moreover, nostalgia proneness, a trait that characterizes how often one experiences nostalgia in everyday life, has been positively linked to the use of effective emotional and instrumental coping strategies (Batcho, 2013). Furthermore, the overall positive effect of nostalgia on affect was supported in two recent meta-analyses (Frankenbach et al., 2021; Leunissen et al., 2021). Building on this evidence, one might expect that compared to recalling an ordinary autobiographical event, nostalgia induction would lead to higher levels of positive affect (*H1*) and lower levels of negative affect (*H2*).

Although nostalgia tends to enhance positive affect, its emotional profile is not univalent. Theoretical and empirical work consistently characterize it as a bittersweet experience, simultaneously blending warmth and social connectedness with longing or loss (Wildschut et al., 2006). This duality implies that nostalgic recall may co-activate positive and negative affective systems, giving rise to a state of affective ambivalence. Supporting this, recent meta-analysis shows that nostalgic inductions increase both happiness and sadness relative to control conditions, resulting in elevated affective ambivalence (Frankenbach et al., 2021; Leunissen et al., 2021). Thus, to fully capture nostalgia’s affective consequences, researchers must move beyond isolated measures of positive and negative affect and directly assess their co-activation as affective ambivalence.

A key challenge in studying nostalgia’s affective complexity lies in how ambivalence is measured. Much of the existing literature operationalizes affective ambivalence as the minimum of ratings for “happy” and “sad” (*e.g.*, Hepper et al., 2024), assuming that co-occurring positive and negative emotions reflect emotional conflict. While this approach sensitively captures the simultaneity of opposing feelings, it disregards their overall intensity: a participant rating both emotions as “2” receives the same ambivalence score as one rating both as “4,” despite the latter reflecting a far more intense emotional experience. To address this limitation, alternative models, such as Griffin’s Similarity-Intensity Model (Thompson et al., 1995), have been proposed. This framework defines ambivalence as a function of both the mean intensity of positive and negative affect and their similarity (*i.e.*, how close the two are in magnitude). By incorporating intensity and balance jointly, it provides a more nuanced index of mixed affective states, better aligned with the bittersweet phenomenology of nostalgia. This view of nostalgia highlights the importance of the induction method, as different procedures may differentially engage these affective systems.

Experimental studies that tested causal links between the experience of nostalgia and affective states used a diverse array of manipulations to induce nostalgia. As recently described in a review by Wildschut and Sedikides (2024), these inductions can be made either by suggesting a vivid recall of past experiences or by presenting sensory stimuli that elucidate nostalgic feelings.

The experimental induction of a vivid recall of past experiences is achieved through an Event Reflection Task (ERT), which asks participants to recall and immerse themselves in a personally meaningful nostalgic memory following a standardized definition (“a sentimental longing for one’s past”; Zhou et al., 2022). Research indicates that the ERT elicits the most profound nostalgic feeling (Wildschut & Sedikides, 2024). However, it has been criticized for limited external validity, as recalling one’s “most nostalgic” memory on demand may not reflect how nostalgia arises spontaneously in daily life (Newman & Sachs, 2020).

An alternative approach includes presentation of sensory stimuli such as visual (pictures, photographs), gustatory (food or smells), and auditory (musical excerpts or song lyrics). Multiple studies that used this approach found that these stimuli effectively induced nostalgia (Cheung et al., 2013; Reid et al., 2015, 2023), but they do not always induce greater positive affect than control conditions (Cheung et al., 2013; Leunissen et al., 2021). Moreover, a key methodological limitation of the sensory induction manipulations lies in the difficulty of dissociating their direct affective impact from the effects specifically attributable to nostalgia induction. Consequently, observed affective responses may be confounded by characteristics inherent to the selected stimulus set (*e.g.*, cheerful music leading both to nostalgia and positive affect; Wildschut & Sedikides, 2024). This limitation might partially explain the effect found in the meta-analysis, where the effect of nostalgia induction on emotion varied by the induction type. For example, nostalgia (compared to control) increased happiness when induced by ERT, but not by music. Further ERT and music inductions increased sadness, but if song lyrics were presented, participants reported decreased sadness (Leunissen et al., 2021).

Consequently, despite extensive experimental investigation, the nature of nostalgia’s influence on affective states remains ambiguous, specifically, whether it elicits uniformly positive, negative, or context-dependent emotional responses. This persistent empirical ambiguity may also indicate that nostalgia’s affective impact is moderated by individual characteristics.

First, age may shape how individuals experience and respond to nostalgia. Although nostalgia effects have been studied across the lifespan, the aforementioned studies were predominantly conducted on student samples (Batcho, 2013; Cheung et al., 2013; Layous et al., 2022; Reid et al., 2015, 2023; Wildschut et al., 2006). As indicated by Leunissen and colleagues (2021), out of 4,659 participants in 41 experiments included in the meta-analysis, 65% were young adults aged less than 35 years. Yet previous studies demonstrated that there are potential age differences in the content and frequency of nostalgia (Batcho, 1995), as well as in sensitivity to nostalgic triggers (Kusumi et al., 2010). Some evidence suggests that nostalgia may have more positive and less ambivalent emotional outcomes among older compared to younger adults (Leunissen et al., 2021). Building on these findings, we hypothesized that (*H3*) the effect of nostalgia induction on affect will be moderated by age, such that older adults will exhibit greater increases in positive affect compared to younger and middle-aged adults.

Second, gender may also shape the emotional outcomes of nostalgic experience. Although not always examined, emerging evidence suggests that men and women differ in their affective responses to nostalgia. For instance, Cheung and collaborators (2013) found that nostalgia induced greater optimism in males than in females. The limited meta-analytic evidence available indicates that gender may serve as a moderator, with females reporting higher levels of sadness and affective ambivalence in nostalgia conditions compared to males (Leunissen et al., 2021). Notably, this gender difference was not observed for happiness, suggesting a complex, emotion-specific pattern of moderation that warrants further investigation. Building on this, we hypothesized that: (*H4A*) the effect of nostalgia induction on affect will be moderated by gender, such that males will exhibit greater increases in positive affect than females; (*H4B*) females will report higher levels of negative affect following nostalgia induction compared to males and (*H4C*) following nostalgia induction, females will report higher levels of affective ambivalence (the simultaneous experience of positive and negative emotions) compared to males.

Third, while some researchers consider nostalgia as a potential intervention for well-being (positive activity; Layous et al., 2022; Layous & Kurtz, 2023) and indicate that nostalgia can buffer low well-being in countries with lower life satisfaction (Hepper et al., 2024), its effects appear conditional on psychological resources. Several competing perspectives yield conflicting predictions. On the one hand, nostalgic experiences could elicit stronger positive emotions in those with higher well-being due to their greater capacity for savoring positive memories (Bryant, 2021). Accordingly, individuals with higher well-being may possess stronger savoring abilities, enabling them to derive more positive emotion from nostalgic memories rather than experiencing sadness over what has been lost. On the other hand, individuals with lower baseline well-being may derive greater benefits from nostalgia due to a compensatory mechanism, as sadness and loneliness were shown to be key triggers of nostalgia (Wildschut et al., 2006). Thus, nostalgic recollection could counteract current emotional deficits by activating comforting past experiences, potentially yielding more pronounced momentary improvements in affect compared to those with higher baseline well-being (the “low base effect”). Alternatively, emerging evidence indicates that nostalgia in daily life may have detrimental effects on well-being for individuals with pre-existing low well-being (*e.g.*, those experiencing loneliness; Newman & Sachs, 2020). This effect is especially pronounced among vulnerable populations. For instance, research conducted with youth in the Gaza Strip found that nostalgia elevated positive affect and social connectedness, but only among those with greater psychological resilience; individuals with low resilience did not experience these benefits (Abu-Rayya et al., 2025). Moreover, nostalgia was found to elevate negative affect among Syrian refugees with low psychological resilience (Wildschut et al., 2019). These complex results suggest that nostalgia’s affective benefits might only be manifest in the absence of concurrent negative life circumstances. Given this evidence, we hypothesized that (*H5*) the effect of nostalgia induction on affect will be moderated by baseline well-being, such that participants with higher levels of well-being will show greater increases in positive affect compared to those with lower levels of well-being.

Finally, the generalizability of findings on nostalgia remains uncertain, as the vast majority of experimental research has been conducted in WEIRD populations (Hepper et al., 2024; Leunissen et al., 2021; Santini et al., 2023) with only occasional evidence from other populations such as Syrian refugees (Wildschut et al., 2019), Gaza youth (Abu-Rayya et al., 2025), and Egyptian Nubians (Agha, 2019). Previous studies on nostalgia in Russia have mainly concerned nostalgia for the Soviet Union (White, 2010) or use of nostalgia in advertising (Holak et al., 2007). Previous research on the role of nostalgia in affective regulation among Russian populations is limited to a small number of observational studies conducted by our group, which positioned nostalgia as a resource for resilience (Bartseva et al., 2025; Vartanyan et al., 2024), and a recent cross-cultural study (Hepper et al., 2024). The latter study identified Russia as one of the least nostalgic or nostalgia-neutral countries, underscoring the need for further empirical inquiry into the function and experience of nostalgia in this cultural context.

The current study addresses these questions in a setting of heightened relevance: the contemporary Russian socio-cultural landscape, marked by rapid change and uncertainty. In such conditions, internally accessible strategies like nostalgia may offer a vital, low-cost tool for emotional regulation. By experimentally testing how nostalgia influences positive affect, negative affect, and affective ambivalence in a demographically diverse Russian-speaking sample, while examining age, gender, and baseline well-being as key moderators, we aim to clarify for whom and under what conditions nostalgia functions as a psychological resource.

Theoretically, this work contributes to cross-cultural models of emotion regulation by testing whether nostalgia’s affective mechanisms are universally robust or culturally contingent. Practically, it informs the potential development of evidence-based, self-administered interventions for non-clinical populations.

**Figure 1.**
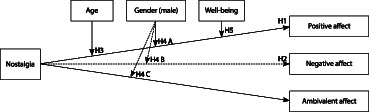
Conceptual model of moderated effects of nostalgia induction on affective outcomes

*Note. Solid lines indicate a positive effect (i.e., higher levels of the moderator are associated with an increase in affective outcome; dashed lines indicate a negative effect (i.e., higher levels of the moderator are associated with a decrease in the affective outcome).*

The present study tests the conceptual model depicted in *Figure 1*.

## Methods

### Participants

The sample included 126 individuals, both men and women, distributed approximately equally across three age groups. After exclusion of individuals who did not experience nostalgia in the nostalgia induction condition (*see Manipulation Check for details*) and participants out of the intended age range, the final sample consisted of 122 people, 64 females, *M*_age_ = 38.63, *SD* = 14.13 (see *Table 1* for *N*s divided by gender and age group).

**Table 1:** Age and Gender Distribution of the Resulting Sample

	**20–35 years**	**36–50 years**	**51–65 years**
Men	23	20	15
Women	23	22	19

### Procedure

Data collection was conducted online by a team of 16 trained interviewers. All interviewers completed a structured training session on the study protocol. They were provided with verbatim scripts for both the nostalgia and neutral induction conditions to ensure consistent delivery. Each interviewer followed a standardized protocol to ensure consistency in the administration of questionnaires and experimental procedures. The research team used quota sampling to ensure balanced representation across gender and age categories, using a snowball sampling technique, starting from research team close contacts (friends and relatives).

Participants completed all tasks and questionnaires during video calls. After providing informed consent, participants were randomly assigned to either the experimental (nostalgia) group or the control (non-nostalgic memory) group. An audio recording was made during the study, to which the participants gave their consent.

All participants first completed the Positive and Negative Affect Schedule (PANAS) to assess their baseline emotional state. Following this, participants in the experimental group were asked to recall and describe a nostalgic memory, while those in the control group were instructed to describe a routine event from the past few days.

To ensure manipulation effectiveness, participants answered control questions designed to verify whether those in the nostalgia group experienced nostalgic feelings (*e.g.*, “I am experiencing nostalgic feelings right now”).

After the experimental manipulation, all participants completed the PANAS again to measure changes in their emotional state. They also completed the WHO-5 Well-Being Index to assess their general level of well-being over the past two weeks. Finally, participants provided demographic information including their age and gender.

**Figure 2.**
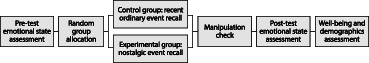
Schematic procedure of the experiment

The entire procedure took approximately 30–45 minutes to complete. Throughout the study, trained interviewers were available online to provide guidance and answer any questions participants might have had about the procedure. *Figure 2* shows the schematic procedure of the experiment.

### Measures

#### Positive and Negative Affect Schedule (PANAS).

The Russian adaptation (Osin, 2012) of the PANAS (Watson et al., 1988) was used to assess participants’ emotional state before and after the experimental manipulation. The scale consists of 20 items, with two 10-item subscales assessing positive affect (PA; “Interested”, “Excited”) and negative affect (NA; “Depressed”, “Upset”). Participants rated the extent to which they experienced each emotion on a 5-point Likert scale ranging from 1 (very slightly or not at all) to 5 (extremely). The Russian version of PANAS has demonstrated good reliability and validity, consistent with the original English version (Osin, 2012). In the current study, the internal consistency was excellent for both subscales at both time points: negative affect pre-test (Cronbach’s α = .915), and post-test (Cronbach’s α = .884), positive affect pre-test (Cronbach’s α = .835), and post-test (Cronbach’s α = .889).

In addition to analyzing PA and NA independently, we computed a composite affective ambivalence (AA) score to capture the simultaneous co-activation of positive and negative emotional systems. This score was calculated using Griffin’s Similarity-Intensity Model of Ambivalence (Thompson et al., 1995) according to the following formula: Ambivalence = [ (PA + NA) / 2 ] − |PA − NA| + C, where PA and NA represent the summed scores from the PANAS subscales, |PA − NA| is the absolute difference between them, and C = 10 was added to ensure non-negative values. This index yields higher scores only when both positive and negative affect are high in intensity and similar in magnitude, capturing strong, balanced mixed affect—the theoretical hallmark of nostalgia’s bittersweet quality. Unlike the minimum-score method (*e.g.*, minimal value for “happy” and “sad”; Hepper et al., 2024), which reflects co-occurrence of specific emotions but ignores overall intensity, the Griffin model quantifies ambivalence at the level of broad affective dimensions. We selected this approach because our hypotheses concerned the generalized co-activation of positive and negative affective systems—and how this co-activation is moderated by stable individual differences (*e.g.*, well-being)—rather than the experience of a single emotion pair. AA scores were computed separately for the pre- and post-manipulation time points.

#### World Health Organization-Five Well-Being Index (WHO-5)

The WHO-5 (The World Health Organization- Five Well-Being Index, n.d.) was used to measure participants’ general well-being. This 5-item questionnaire asks participants to rate how they have been feeling over the last two weeks on a 6-point Likert scale from 0 (at no time) to 5 (all of the time). Example item: “I have felt cheerful and in good spirits”. The raw score, ranging from 0 to 25, is multiplied by 4 to give the final score, with 0 representing the worst well-being and 100 the best well-being.

The official Russian-language version of the WHO-5 scale was developed by WHO. The scale has good internal consistency: Cronbach’s α coefficients ranging from .82 to .95. In the present study, the WHO-5 demonstrated good internal consistency (Cronbach’s α = .862).

The WHO-5 was administered after the experimental manipulation. Although a baseline assessment would be ideal for establishing temporal precedence, we opted to administer it post-intervention to minimize participant burden and reduce the risk of testing fatigue or carryover effect, which could compromise response quality on our primary affect measures. Critically, the WHO-5 is explicitly designed as a retrospective measure of well-being over the preceding two weeks, thereby capturing a relatively stable, trait-like construct that largely predates the experimental session. Taken together, these psychometric properties justify treating post-session WHO-5 scores as a reasonable proxy for pre-intervention well-being.

#### Nostalgia Manipulation

Participants in the experimental group performed the Event Reflection Task (Wildschut & Sedikides, 2024) and were asked to recall and describe a nostalgic event from their past, while those in the control group were instructed to recall and describe a routine event from the past few days. The exact instructions for the experimental group were as follows: “Now you are asked to complete a task related to a nostalgic memory. Nostalgia is a ‘sentimental longing for the past’ — for example, for a childhood camp, school friends, or family trips. You will need to recall an event from the past that evokes the greatest nostalgia in you. Take a few minutes to think about this nostalgic event, immerse yourself in it and the feelings it evokes in you. Then tell me about this event as much as you consider necessary.”

For the control group, the instructions were: “You will need to complete a task related to your memories. Please recall and describe what happened to you three days ago. Describe where you were and what you were doing during that day, what emotions you experienced. You can think about it a little and then tell as much as you consider necessary.”

#### Manipulation Check

To ensure the effectiveness of the nostalgia manipulation, participants in both the experimental and control groups completed a three-item manipulation check immediately after the recall task, adopted from a previous study (Cheung et al., 2013). All items were rated on a 5-point Likert scale (1 = not at all to 5 = very much). The items were: “I am experiencing nostalgic feelings right now”; “I have nostalgic thoughts right now”; and “I feel nostalgia at this moment.” Internal consistency for the three items in the current study was excellent (Cronbach’s *α =* .9)

Participants in the nostalgia condition were required to demonstrate a clear subjective experience of nostalgia. To ensure this, we applied an exclusion criterion based on the scale’s face validity: a total score below 9 (out of a maximum of 15) indicates that the participant responded with “3 = neutral” or lower (*i.e.*, not at all, slightly, or moderately) on at least one of the three key items, thereby failing to affirm the presence of nostalgic feeling, thought, or experience. This threshold was chosen because a score of 9 corresponds to the lowest total possible when all three items are rated at the midpoint (“neutral”); any lower total implies active disagreement or absence of the nostalgic state. Following this criterion, three participants from the nostalgia condition were excluded, ensuring that all retained participants reported at least moderate agreement across all items measuring state nostalgia.

The final analytic sample showed a significant difference in manipulation check scores between conditions: *M* = 12.87 (*SD* = 1.86) in the nostalgia group versus *M* = 9.68 (*SD* = 3.39) in the control group, *Mann–Whitney U* = 2917, *p* < .001, confirming the success of the experimental manipulation.

### Statistical Approach

To examine the effects of measurement time (pre- vs post-manipulation), experimental condition (nostalgia induction vs. neutral memory), and individual differences (age, gender, well-being) on affect (PA, NA, and AA), we fitted a linear mixed-effects model using the lmer function from the lme4 package (version 1.1.35.3; Bates et al., 2014) in RStudio (R version 4.3.3). The model included fixed effects for Time (within-participant), Condition (between-participants), and their interaction, as well as interactions between Condition and individual difference variables (age, gender, and well-being). These interactions tested whether the effect of experimental condition on affect varied as a function of these participant characteristics.

A random intercept for participant ID was included to account for repeated measures within individuals and to account for baseline differences between individuals. The model was estimated using restricted maximum likelihood (REML).

All continuous predictors (age, WHO5) were mean-centered prior to analysis to facilitate interpretation of interaction terms. Model assumptions (normality and homoscedasticity of residuals) were checked visually using diagnostic plots and were found to be reasonably met.

## Results

Descriptive statistics for all study variables in the two groups are available in *Table 2*. Mann-Whitney U tests were conducted to compare the control and experimental groups. The results indicated greater pre-intervention positive affect in the control group compared to the nostalgia induction group, with a small effect size (rank-biserial *r* = –.24, 95% CI [–.42, –.04]). This baseline difference is not a confound for testing the intervention effect, as our primary models use each participant as their own control (via the random intercept) and tests the change in affect from pre- to post-intervention.

**Table 2:** Descriptive Statistics of the Study Variables in Two Groups

	**Control condition (*N* = 59)**			**Nostalgia condition (*N* = 63)**			**Group comparison**	
**Variable**	**Mean**	***SD***	**Min-Max**	**Mean**	***SD***	**Min-Max**	**Mann-Whitney U**	***p***
Negative Affect (Pre-test)	16.39	6.51	10–41	17.48	8.12	10–45	1896	.85
Negative Affect (Post-test)	14.34	5.33	10–32	15.11	6.21	10–36	1947	.65
Positive Affect (Pre-test)	32.07	5.92	15–46	29.52	6.15	15–47	1409.5	.02
Positive Affect (Post-test)	31.97	6.82	15–48	30.65	7.14	11–47	1668.5	.33
Ambivalent affect (Pre-test)	17.26	8.36	3.5–41	18.88	9.24	5.5–40	2016	.42
Ambivalent affect (Post-test)	15.02	8.54	1–42	16.01	8.70	2.5–34	2001.5	.46
Well-being (WHO-5)	51.25	19.83	0–80	48.44	20.19	0-88	1717.5	.47

**Figure 3.**
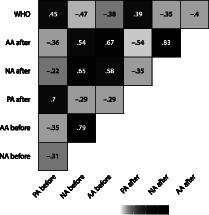
Correlations between study variables

Further, we inspected correlation coefficients between study variables (*Figure 3*). All correlations were significant at the *p* < .05 level. Well-being showed moderate correlations with measures of affect, while the strongest relationships were between pre-and post-intervention scores for positive, negative, and ambivalent affect.

### Positive Affect

To explore the relationship between nostalgia, affect, and individual differences, linear mixed-effect regression analysis was conducted for positive affect (*Table 3*).

**Table 3:** Results of the Mixed-Effect Linear Regression for Positive Affect after Memory Induction

**Predictor**	**B**	**SE**	**β**	**95% CI**	***p***
(Intercept)	28.75	.99	–.34	26.8 – 30.71	< **.001**
Time (after)	1.17	.85	.18	–.52 – 2.85	.17
Condition (ordinary)	2.4	1.54	.36	–.64 – 5.43	.12
Time (after) × Condition (ordinary)	–1.65	1.33	–.25	-4.27 – .97	.21
Time (before) × Condition (nostalgia) × Age	–.02	.05	–.04	–.12 – .08	.73
Time (after) × Condition (nostalgia) × Age	–.05	.05	–.12	–.16 – .05	.29
Time (before) × Condition (ordinary) × Age	–.01	.06	–.02	–.13 – .11	.86
Time (after) × Condition (ordinary) × Age	–.08	.06	–.17	–.2 – .04	.19
Time (before) × Condition (nostalgia) × Gender (male)	2.17	1.54	.33	–.86 – 5.2	.16
Time (after) × Condition (nostalgia) × Gender (male)	2.07	1.54	.31	–.96 – 5.1	.18
Time (before) × Condition (ordinary) × Gender (male)	1.28	1.63	.19	–1.94 – 4.49	.43
Time (after) × Condition (ordinary) × Gender (male)	2.13	1.63	.32	–1.09 – 5.34	.19
Time (before) × Condition (nostalgia) × WHO	.1	.04	.31	.03 – .18	**.007**
Time (after) × Condition (nostalgia) × WHO	.12	.04	.37	.05 – .2	**.001**
Time (before) × Condition (ordinary) × WHO	.16	.04	.48	.08 – .24	< **.001**
Time (after) × Condition (ordinary) × WHO	.14	.04	.43	.06 – .22	< **.001**
**Random Effects**					
σ^2^					13.24
τ_00 ID_					22.38
ICC					.63
N _ID_					122
Observations					244
Marginal *R*^2^ / Conditional *R*^2^				.	216 / .708
AIC					1.545.281

As shown in *Table 3*, fixed effects explained around 22% of the variance in PA (marginal *R^2^*), while fixed and random effects together explained 70% (conditional *R^2^*), indicating substantial individual-level variability.

The main effect of time (pre- to post-experimental manipulation) was non-significant, suggesting no significant change in PA in both conditions. The main effiect of condition (nostalgic vs. control) was also non-significant, suggesting no overall difference in PA between groups. The interaction between time and condition was non-significant, thus rejecting *H1*.

**Figure 4.**
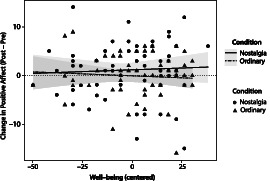
Well-being predicts PA change after ordinary memory and induced nostalgia

Among moderators, only significant time × condition × WHO-5 interactions occurred, revealing that well-being moderates the effect of condition on change in PA (*Figure 4*). Specifically, the positive significant coefficient indicates that the effect of the nostalgia induction on positive affect over time was stronger for individuals with higher initial well-being: for every one-unit increase in WHO-5 score, the nostalgia intervention produced an additional 0.12-unit increase in positive affect from pretest to post-test, thus partially supporting *H5*. Well-being was also associated with changes in PA in the control group.

*Figure 4* visualizes the effect of interaction between experimental condition and well-being on changes in PA. The horizontal dashed line represents zero change (Post – Pre = 0), serving as a reference for interpreting the direction of effects. Among participants with lower baseline well-being, PA change scores clustered around zero in both the nostalgia (solid line) and control (dashed line) conditions. However, at higher levels of well-being, the conditions diverged: participants in the nostalgia condition showed a slight increase in PA (regression line above zero), whereas those in the control condition remained near the no-change threshold.

This asymmetry in the control group (improvement at low baseline and decline at high baseline) is consistent with regression to the mean (RTM), a statistical tendency for extreme scores to revert toward the mean on retest. To test this, we correlated pre-test PA with PA change scores (Post – Pre) within each condition. RTM would be manifest as a strong negative correlation. However, the results showed only a small, significant correlation in the nostalgia condition (*r* = –.25, *p* = .038) and a non-significant correlation in the control condition (*r* = –.17, *p* = .197). These modest effects suggest that RTM played, at most, a minor role of the observed patterns.

No significant three-way interactions were found for age or gender, failing to support *H3* and H4A.

Finally, as a robustness check, we re-estimated the model using centered self-reported nostalgia scores (from the manipulation check) in place of the experimental condition, including all participants. This analysis yielded no significant main, interaction, or mediation effects, reinforcing that the observed moderation by well-being is tied to experimental assignment, not subjective nostalgia intensity.

### Negative Affect

As shown in *Table 4*, for NA, fixed effects explained 27% of the variance (marginal *R^2^*), while fixed and random effects together explained 68% (conditional *R^2^*), again indicating substantial individual-level variability.

**Table 4:** Results of the Mixed-Effect Linear Regression for Negative Affect after Memory Induction

**Predictor**	**B**	**SE**	**β**	**95% CI**	*P*
(Intercept)	19.14	.97	.49	17.23 – 21.05	**<.001**
Time (after)	–3.03	.92	–.45	–4.83 – –1.22	**.001**
Condition (ordinary)	–3.88	1.51	–.58	–6.85 – –.9	**.011**
Time (after) × Condition (ordinary)	1.72	1.43	.26	–1.08 – 4.53	.23
Time (before) × Condition (nostalgia) × Age	–.1	.05	–.2	–.2 – .01	.06
Time (after) × Condition (nostalgia) × Age	–.08	.05	–.16	–.18 – .03	.14
Time (before) × Condition (ordinary) × Age	.03	.06	.06	–.09 – .14	.62
Time (after) × Condition (ordinary) × Age	–.05	.06	–.11	–.17 – .06	.36
Time (before) × Condition (nostalgia) × Gender (male)	–4.63	1.51	–.69	–7.6 – –1.66	**.002**
Time (after) × Condition (nostalgia) × Gender (male)	–2.87	1.51	–.43	–5.84 – .1	.06
Time (before) × Condition (ordinary) × Gender (male)	2.56	1.6	.39	–.56 – 5.74	.11
Time (after) × Condition (ordinary) × Gender (male)	1.03	1.6	.15	–2.12 – 4.18	.52
Time (before) × Condition (nostalgia) × WHO	–.13	.04	–.38	–.2 – –.06	**.001**
Time (after) × Condition (nostalgia) × WHO	–.09	.04	–.28	–.17 – –.02	**.012**
Time (before) × Condition (ordinary) × WHO	–.21	.04	–.61	–.28 – –.13	**<.001**
Time (after) × Condition (ordinary) × WHO	–.09	.04	–.27	–.17 – –.01	.02
**Random Effects**					
σ^2^					15.24
τ_00 ID_					18.99
ICC					.55
N _ID_					122
Observations					244
Marginal *R*^2^ / Conditional *R*^2^					.273 / .676
AIC					1.551.565

**Figure 5.**
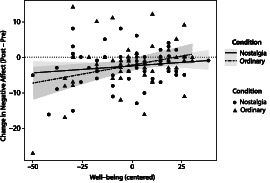
Well-being predicts NA change after ordinary memory and induced nostalgia

NA scores decreased significantly from pre- to post-manipulation across both conditions, but the condition × time interaction was non-significant, thus rejecting *H2*. At baseline, those in the control condition reported significantly lower NA scores compared to the nostalgic condition, suggesting that individuals in the nostalgic condition may have started with greater emotional distress.

There was a moderation effect of gender. Males in the nostalgia condition reported lower NA than females before the intervention, but not after nostalgia induction.

Finally, well-being significantly moderated the effect of condition on NA change (*β* = –.28, *p* = .012), partially supporting *H5*. *Figure 5* illustrates this pattern. Values below the zero-change line indicate a reduction in NA. Among participants with lower well-being, both conditions produced reductions in NA (regression lines below zero), with the control condition showing larger decrease. However, at higher levels of well-being, the patterns diverged: the nostalgia condition stabilized NA (line approaching zero), whereas the control condition was associated with a slight increase in NA (line crossing above zero).

Crucially, the well-being × condition interaction was not significant, indicating that the moderating role of well-being did not differ meaningfully between conditions. In other words, higher well-being was associated with greater NA reduction, but this effect was not uniquely enhanced by nostalgia.

As a robustness check, we re-estimated the model using centered self-reported nostalgia scores instead of experimental condition. In this analysis, nostalgia intensity was a significant positive predictor of NA (*β* = .73, *p* = .007), indicating that participants who felt more nostalgic also reported higher overall levels of negative affect. However, the time × nostalgia interaction was non-significant, confirming that change in NA was not driven by subjective nostalgia intensity. A significant nostalgia × well-being interaction at pre-test (higher well-being linked to lower NA among highly nostalgic individuals) disappeared post-test, further suggesting that well-being shaped baseline affective states but not intervention-related change.

### Ambivalent Affect

As shown in *Table 5*, for AA, fixed effects explained 22% of the variance (marginal *R^2^*), while fixed and random effects together explained 68% (conditional *R^2^*), again indicating substantial individual-level variability. AA scores decreased significantly from pre- to post-intervention across both conditions. The conditions did not differ in ambivalent affect.

**Table 5:** Results of the Mixed-Effect Linear Regression for Ambivalent Afffect after Memory Induction

Predictor	B	SE	β	95% CI	*p*
(Intercept)	20.53	1.32	.42	17.93 – 23.13	**<.001**
Time (after)	–3.45	1.19	–.39	–5.8 – –1.1	**.004**
Condition (ordinary)	–3.11	2.05	–.35	–7.16 – .93	.13
Time (after) × Condition (ordinary)	1.67	1.86	.19	–1.98 – 5.33	.37
Time (before) × Condition (nostalgia) × Age	–.1	.07	–.16	–.24 – .03	.14
Time (after) × Condition (nostalgia) × Age	–.1	.07	–.16	–.24 – .04	.15
Time (before) × Condition (ordinary) × Age	–.04	.08	–.07	–.2 – .11	.58
Time (after) × Condition (ordinary) × Age	–.06	.08	–.09	–.21 – .1	.48
Time (before) × Condition (nostalgia) × Gender (male)	–4.43	2.05	–.5	–8.47 – –.39	**.03**
Time (after) × Condition (nostalgia) × Gender (male)	–3.24	2.05	–.37	–7.27 – .8	.11
Time (before) × Condition (ordinary) × Gender (male)	.42	2.17	.05	–3.86 – 4.71	.85
Time (after) × Condition (ordinary) × Gender (male)	–.58	2.17	–.07	–4.86 – 3.71	.79
Time (before) × Condition (nostalgia) × WHO	–.07	.05	–.17	–.17 – .03	.15
Time (after) × Condition (nostalgia) × WHO	–.14	.05	–.31	–.24 – –.04	**.008**
Time (before) × Condition (ordinary) × WHO	–.24	.05	–.55	–.35 – –.13	**<.001**
Time (after) × Condition (ordinary) × WHO	–.18	.05	–.42	–.29 – –.08	**.001**
**Random Effects**					
σ^2^					25.85
τ^00 ID^					37.38
ICC					.59
N ^ID^					122
Observations					244
Marginal *R*^2^ / Conditional *R*^2^					.221 / .682
AIC					1.684.351

**Figure 6.**
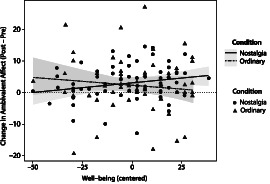
Well-being predicts AA change after ordinary memory and induced nostalgia

Male participants in the nostalgia condition reported lower AA before the intervention. No difference in AA between males and females was found after nostalgia induction.

Well-being significantly moderated the effect of condition on AA change (supporting *H5*). As *Figure 6* shows, the pattern diverged meaningfully across conditions. Among participants with lower well-being, the control condition was associated with an increase in AA (dashed line above zero), whereas the nostalgia condition produced no reliable change (solid line near zero). Conversely, at higher levels of well-being, this pattern reversed: nostalgia recall was linked to increased AA (solid line rising above zero), while the control condition approached stability (dashed line converging on zero).

In the control condition (dashed line), the pattern was qualitatively different: individuals with low well-being showed a small reduction in AA, but those with high well-being actually exhibited a slight increase in ambivalence after recalling an ordinary memory. This reversal implies that neutral autobiographical reflection may disrupt emotional clarity among those who typically experience low ambivalence.

As a robustness check, we re-estimated the model using centered self-reported nostalgia scores instead of experimental condition. The main effect of time remained significant, but nostalgia intensity was not associated with higher baseline AA, and the time × nostalgia interaction was non-significant. Moreover, no higher-order interactions with well-being, age, or gender reached significance.

## Discussion

The present study examined the emotional impact of nostalgia induction compared to a neutral memory recall condition, with a focus on changes in positive affect (PA), negative affect (NA), and affective ambivalence (AA). We also examined whether these effects were moderated by age, gender, and well-being (as measured by WHO-5).

*H1* and *H2* predicted that participants in the nostalgia condition would experience a greater increase in positive affect and greater decrease in negative affect compared to the daily recollection of life events. Contrary to previous literature (Frankenbach et al., 2021; Leunissen et al., 2021) which has documented overall boosts in positive affect following nostalgic recall, the nostalgia induction did not lead to increases in positive affect or decreases in negative affect when compared to the control condition in our sample. While this could reflect nostalgia’s bittersweet nature (Batcho, 2013) — where positive emotions coexist with loss — the absence of changes in ambivalence scores rules out this explanation and casts some doubts on the previously suggested overall positivity of nostalgic experiences (Frankenbach et al., 2021; Leunissen et al., 2021), especially induced under controlled conditions (Newman et al., 2020). Our study’s online format (data were collected in 2024) created a unique methodological context. It lacked the high degree of control and potential stress of a laboratory environment, yet the requirement to recollect a nostalgic memory on demand remained an artificial task, one that does not fully mirror the contextual nature of nostalgic experiences in daily life. Therefore, our findings contribute to a more critical perspective, indicating that nostalgia’s positive effects are not universal and may be dependent on the context of its induction.

An important context for interpreting the ambivalence findings is the baseline affective profile of our non-clinical sample, where positive affect scores were significantly higher than negative affect scores across conditions. This pattern is typical in community samples and does not invalidate the ambivalence measure. On the contrary, the Griffin formula is mathematically sensitive to this asymmetry; it inherently provides low ambivalence scores when one affect system strongly dominates and higher scores only when both systems are concurrently active. Therefore, the observed experimental effects on AA reflect meaningful changes in this mixed affective state, supporting the hypothesis that nostalgia specifically engages and then modulates emotional conflict.

The present study revealed a reduction in negative and ambivalent affect not only in the nostalgia condition but also in the control condition. This finding might have several potential explanations. First, the core procedure of recalling an autobiographical memory, irrespective of its specific emotional valence, may in itself possess regulatory power. This act of reflection could promote a state of mindfulness and emotional processing, leading to a generalized reduction in negative affect. Alternatively, it is possible that the memories that participants self-selected as “ordinary” were, in fact, neutral-positive or otherwise personally meaningful, thereby producing a similar, albeit potentially weaker, regulatory effect compared to nostalgia. To elucidate the nature of this effect, future research should directly compare the emotional, thematic, and linguistic content of nostalgic and ordinary memories. Such an analysis will be crucial for pinpointing the unique components that differentiate nostalgia from other forms of positive autobiographical thought.

This result could also be explained by slightly higher initial negative affect in the nostalgia condition group, meaning that there was more negative affect to reduce in the nostalgia group compared to the neutral memory group, which nostalgia apparently failed to overcome. Studies that allow for better balance between groups in their initial levels of negative affect (and other traits) or studies that implement negative emotions induction (*e.g*., with a sad video) before nostalgia induction, to unify starting ground for both groups, are needed in order to investigate this effect further.

The Russian context adds further nuance. Hepper and collaborators (2024) identified Russia as a “nostalgia-neutral” culture, and our sample may engage with nostalgia differently than WEIRD populations. The lack of universal positivity may reflect this cultural backdrop, where nostalgic reflection on the past does not automatically invoke comfort, but may evoke ambivalence about national or personal history. Thus, our findings caution against universalizing Western models of nostalgia as a purely adaptive resource.

Although the main effects of nostalgia were limited, we identified well-being as a potential moderator of its effects, supporting *H5*. Specifically, well-being significantly moderated the effect of intervention on positive affect, with participants higher in well-being showing greater positive affect after nostalgia induction. This interaction suggests that nostalgia may have emotional benefits, but primarily for individuals already at a higher baseline level of well-being. The same pattern was found for negative affect: individuals with higher well-being were somewhat buffered against negative emotional responses after nostalgia induction. Individuals with higher well-being also demonstrated higher affective ambivalence after nostalgia induction. These results align with prior research highlighting that pre-existing psychological resources are a critical moderator of autobiographical memory’s impact on mood. For instance, individuals with depression derive less positive affect from happy memories and experience intensified negative affect from sad memories (Kim & Yoon, 2020). Similarly, the beneficial effects of nostalgia were found to be contingent on attachment security; only participants with low insecurity showed mood improvements, while those with high insecurity did not (Cavanagh et al., 2015).

Together these findings align with the vantage sensitivity framework (De Villiers et al., 2018), which posits that individuals vary in their responsiveness to positive interventions due to differences in environmental sensitivity. The moderation effect of well-being suggests that pre-existing psychological resources may enhance sensitivity to positive stimuli described in the model. Specifically, those with higher well-being exhibited amplified positive affect and affective ambivalence, reduced negative affect after nostalgia, consistent with the idea that they are better equipped to extract emotional value from positive experiences. Conversely, individuals with lower well-being showed muted responses, possibly reflecting lower sensitivity to the intervention’s benefits. This pattern underscores that nostalgia’s efficacy is not uniform, but hinges on an individual’s capacity to process and benefit from positive emotional cues—a core tenet of vantage sensitivity. Future research could explicitly measure environmental sensitivity to test whether it accounts for the observed well-being moderation.

Notably, our findings are more consistent with the vantage sensitivity framework than with the differential susceptibility hypothesis (Belsky & Pluess, 2009). While differential susceptibility posits that certain individuals are more responsive to both positive and negative environmental influences, our results revealed an asymmetric pattern: individuals with higher baseline well-being derived greater emotional benefits from nostalgia induction, whereas those with lower well-being showed neither heightened gains nor increased vulnerability. This suggests that well-being functions primarily as a facilitator of positive intervention effects rather than a broad marker of environmental susceptibility. Such a distinction underscores the importance of considering individual differences in psychological resources when designing and implementing emotion-focused interventions.

In line with previous findings (Santini et al., 2023), there was no consistent evidence supporting H3, which predicted that older adults would benefit more in terms of positive affect. Age did not significantly moderate the effect of nostalgia on changes in positive, negative or ambivalent affect. This suggests that while nostalgia has been found to be more prominent for older adults (Batcho, 1995; Kusumi et al., 2010), its capacity to enhance positive emotions may be comparable across age groups in the context of this brief experimental induction.

Similarly, *H4A-H4C*, predicting that males would show different patterns of positive, negative, and ambivalent affect than females, was not supported by the data. Gender did not significantly moderate changes in positive, negative or ambivalence affect after nostalgia induction.

Together these findings suggest that nostalgia’s emotional effects are complex and conditional. While it does not universally boost positive affect or reduce negative affect, its benefits become more evident when considering individual differences. Well-being emerged as a potential moderator across all affective domains, suggesting that nostalgia functions more effectively as an emotional resource when individuals already possess adequate psychological resources. Gender and age did not significantly shape emotional changes from pre- to post-intervention after a brief nostalgia induction.

Practically, these results suggest that nostalgia-based interventions might be most effective for individuals with relatively high baseline well-being. Future research should aim to identify the thresholds of well-being at which nostalgia is most safely and effectively applied.

## Conclusion

In sum, the present study found limited overall emotional benefits of nostalgia induction but revealed potential moderating effects of well-being. Our findings suggest that nostalgia may function less as a universal mood-enhancer and more as a personalized regulatory tool whose effectiveness depends on the individual’s psychological profile. For example, individuals with higher well-being may have more emotional benefit or resilience from nostalgic reflection, while individuals with lower well-being might experience greater ambivalence or limited gains.

These results have important theoretical implications for understanding the role of nostalgic experiences in the formation of emotional states. The results of the work may contribute to the understanding of the regulatory function of nostalgia and its role in coping strategies, and contribute to the development of mental health support strategies based on the use of nostalgia as a resource for resilience.

## Limitations

A key methodological limitation of this study is that well-being, assessed using the WHO-5, was measured after the experimental manipulation rather than at baseline. Although the WHO-5 is explicitly designed to capture relatively stable subjective well-being over the preceding two weeks and has demonstrated high test–retest reliability over a 7-day interval (Yang et al., 2023), we cannot entirely rule out the possibility that the nostalgia induction influenced participants’ retrospective well-being reports. Such reverse causality would threaten the internal validity of interpreting well-being as a pre-existing moderator of intervention effects. To address this concern empirically, we conducted a supplemental analysis comparing post-intervention WHO-5 scores between the nostalgia and control conditions. The absence of a significant difference between groups (*t*(120.46) = -.712, *p* = .48) provides preliminary evidence that the manipulation did not systematically alter well-being ratings, supporting our interpretation of WHO-5 as a proxy for pre-existing differences. Nevertheless, to definitively establish temporal precedence and rule out confounding, future studies should administer the WHO-5 prior to any experimental intervention.

Second, although our sample size (*N* = 122) was sufficient to detect medium-to-large main effects consistent with prior nostalgia research, it provided limited power to detect smaller or more complex effects, such as three-way interactions. A post-hoc sensitivity analysis indicated that we could reliably detect interactions explaining ≥7% of additional variance (*η^2^_p_* ≥ .07), a threshold met by our well-being moderation effects. However, for non-significant interactions (*e.g.*, age, gender), confidence intervals were wide and included both null and substantively meaningful effects. Thus, these null findings should be interpreted as inconclusive, not as evidence of absence. Furthermore, the use of quota and snowball sampling methods constrains the generalizability of the findings. While quota sampling ensured diversity on key demographics, and snowball sampling aided recruitment, these non-probability methods introduce the potential for selection bias and reduce external validity. The sample may not fully represent the broader population, as it could over-represent certain social networks or community groups. Future research aimed specifically at investigating these higher-order interactions may require larger randomly selected samples to achieve adequate statistical power.

Third, our design did not account for potential interviewer effects. Sixteen different interviewers conducted the sessions, and we did not record interviewer IDs, preventing us from statistically modelling this source of clustering (*e.g.*, with a random intercept for interviewer). While we implemented rigorous procedural controls (including standardized scripts, training, and supervised practice, to ensure consistent delivery), we cannot rule out that unmeasured variance between interviewers influenced the results. This may affect the generalizability and precision of our effect estimates. Future research using interviewer-administered interventions must record interviewer identifiers and include them as a random factor in the statistical model to partition and control for this variance, thereby strengthening internal validity.

Fourth, we relied on the Event Reflection Task (ERT) for nostalgia induction, a method that has been critiqued for potential constraints on external validity (Newman et al., 2020). Further, the use of a brief, one-time induction may not have been strong enough to elicit large emotional changes, especially for those with low engagement or low baseline nostalgia proneness. To address these concerns, future research should pursue two key directions: (1) replication of the present findings using alternative nostalgia induction techniques (*e.g.*, sensory stimuli such as visual or auditory cues) and (2) direct empirical comparisons between the ERT and other induction methods to assess their relative efficacy in eliciting nostalgic intensity and influence affective states. Research that assesses whether turning to nostalgia repeatedly across an extended period (week, months, year) is also needed, as previous studies showed that people who are prone to nostalgia (*i.e.*, high on nostalgia proneness) could experience nostalgia several times a week and the effects of such a regular dose of nostalgia are yet to be investigated. Such methodological refinements would strengthen the generalizability of the results and clarify the role of induction-specific effects in nostalgia research.

Finally, while affective ambivalence was measured, future research might benefit from using qualitative or open-ended data to capture the emotional complexity of nostalgic memories more richly.
